# Transcutaneous auricular vagus nerve stimulation for major depressive disorder: current evidence and future research directions

**DOI:** 10.3389/fpsyt.2026.1788611

**Published:** 2026-03-11

**Authors:** Jifei Sun, Deqiang Gao, Jing Zhang, Hongwei Liu, Yuan Zhou, Chenjie Ma, Xiaojian Zhang, Shuqing Liu, Jingxue Zhao, Chunbo Hao, Xue Xiao

**Affiliations:** 1 Department of Neurology, Shunyi Hospital, Beijing Hospital of Traditional Chinese Medicine, Beijing, China; 2 Guang’anmen Hospital, China Academy of Chinese Medical Sciences, Beijing, China; 3 Department of Pain Management, Beijing Fengtai You'anmen Hospital, Beijing, China; 4 Department of Psychiatry and Psychology, Beijing Tsinghua Changgung Hospital, Beijing, China

**Keywords:** gut-brain axis, major depressive disorder, narrative review, neuroimaging, neuromodulation, transcutaneous auricular vagus nerve stimulation

## Abstract

Major depressive disorder (MDD) causes a significant global disease burden, yet existing pharmacological treatments are often limited by side effects and poor adherence. Transcutaneous auricular vagus nerve stimulation (taVNS), a promising non-invasive neuromodulation technique, offers a safe and accessible therapeutic alternative. This narrative review synthesizes current clinical evidence, explores potential mechanisms of action, and outlines future research directions for taVNS in MDD. Clinical studies consistently indicate that taVNS significantly alleviates depressive symptoms. Its therapeutic effects are mediated through multiple pathways, including anti-inflammatory regulation, autonomic modulation, neurotransmitter restoration, functional brain network reorganization, and the gut-brain axis. Despite these promising findings, clinical translation is hindered by methodological heterogeneity, small sample sizes, and a lack of long-term follow-up data. To advance clinical application, future research should prioritize establishing standardized stimulation protocols to reduce methodological heterogeneity, integrating multi-omics approaches to systematically decipher the “neuro-immune-endocrine-gut-brain” regulatory axis, and leveraging machine learning algorithms to identify multimodal predictive biomarkers for precision medicine. By integrating these advanced approaches, taVNS has the potential to become a robust, first-line therapeutic option for depression.

## 1 Introduction

Major Depressive Disorder (MDD) is characterized by persistent low mood, anhedonia, and cognitive impairment (1). Epidemiological data indicate a global prevalence of approximately 3.8%, with a higher incidence in females. In China, the lifetime prevalence is estimated at 6.8% (2). By 2030, MDD is projected to become the leading cause of global disease burden, significantly impairing daily functioning and quality of life (3, 4). Consequently, identifying effective therapeutic strategies remains a critical priority.

Currently, first-line clinical intervention for MDD primarily relies on antidepressant medications. However, such treatment strategies are often associated with issues including risk of drug dependence, noticeable side effects, and poor patient medication adherence (5). As an adjunctive therapy for treatment-resistant depression (TRD), vagus nerve stimulation (VNS) was approved by the U.S. Food and Drug Administration in 2005 for alleviating depressive and anxiety symptoms (6). Nevertheless, its invasive implantation procedure, high treatment costs, and potential risks of surgery-related infections have limited its broad clinical application.

TaVNS is a non-invasive neuromodulation technique that achieves therapeutic effects by electrically stimulating the vagus nerve branches in the auricular concha region, effectively overcoming the invasive limitations of traditional VNS (7). Existing clinical studies indicate that taVNS significantly improves symptoms such as depression, anxiety, and sleep disorders, with efficacy comparable to that of VNS (8–10). Moreover, it offers notable advantages including non-invasiveness, ease of operation, high safety, and lower economic burden, demonstrating promising clinical application prospects. While repetitive transcranial magnetic stimulation (rTMS) is a well-established non-invasive treatment, it typically relies on bulky equipment and requires administration by trained professionals in hospital settings, which limits its accessibility for daily or long-term maintenance therapy (11). Similarly, although transcranial direct current stimulation (tDCS) offers portability, it involves direct electrical stimulation of the cranium and is frequently associated with adverse effects such as skin irritation, itching, or sensations of burning at electrode sites (12). In contrast, taVNS occupies a unique clinical niche. It modulates central nervous system function via peripheral nerve stimulation, thereby avoiding the safety risks associated with direct intracranial currents. Consequently, taVNS demonstrates a superior safety profile with minimal side effects and high patient tolerability. Its distinctive advantages—compact portability, cost-effectiveness, and suitability for self-administered home use—make it an ideal intervention for broadening access to mental healthcare and facilitating long-term disease management outside of clinical settings. Several recent studies suggest that this technique can significantly reduce clinical depression ratings (13–15). However, it should be noted that current evidence is largely based on small-sample, short-term trials. There is still a lack of high-quality studies systematically validating the consistent efficacy and neurobiological mechanisms of taVNS for treating MDD. This review aims to synthesize existing clinical evidence to comprehensively evaluate the effectiveness and safety of taVNS in treating depression, explore its potential mechanisms of action, and provide theoretical support and practical guidance for promoting the clinical translation of this technology.

MDD is a complex disorder arising from the interplay of genetic vulnerability, neurochemical alterations, endocrine-immune abnormalities, and psychosocial factors (16; 17). Its pathogenesis involves multilevel dysregulation across molecular, cellular, neural circuit, and systemic domains. A key mechanism is deficiency in monoaminergic neurotransmitter systems, including reduced levels of serotonin (5-HT), norepinephrine (NE), and dopamine (DA). This deficiency impairs emotion regulation, motivation, and cognitive control (4, 18). Hyperactivation of the hypothalamic-pituitary-adrenal (HPA) axis leading to excessive glucocorticoid release (19), which exerts neurotoxic effects on brain regions including the hippocampus and prefrontal cortex, resulting in neuronal atrophy, diminished synaptic plasticity, and further suppression of monoamine synthesis, thereby sustaining a vicious cycle (20). Neuroimmune abnormalities characterized by systemic low-grade inflammation and elevated pro-inflammatory cytokines (21), which disrupt neurotransmitter metabolism, reduce neurotrophic support, and compromise blood-brain barrier integrity (22). Alterations in brain structure and functional networks, manifesting as volumetric reductions in the prefrontal cortex, hippocampus, and amygdala, as well as functional connectivity (FC) disturbances such as hyperactivation of the default mode network (DMN) (23) and hypo-connectivity within the central executive network (CEN) (24) and salience network (SN) (25). Dysregulation of neurotrophic signaling, particularly decreased expression of brain-derived neurotrophic factor (BDNF), impairing neuronal survival and synaptic function (26). Genetic and epigenetic mechanisms, such as polymorphisms in genes including 5-HTTLPR and BDNF Val66Met, as well as stress-induced epigenetic modifications like DNA methylation and histone alterations, mediate long-term behavioral and physiological adaptations by modulating gene expression (27, 28). These interconnected mechanisms collectively lead to widespread dysfunction in emotional regulation, stress response, and higher-order cognitive processes, ultimately culminating in the persistent emotional, cognitive, and somatic symptoms characteristic of major depressive disorder.

Although several reviews have addressed taVNS for depression in recent years (6, 29, 30), most have focused primarily on clinical efficacy or isolated biological mechanisms, often lacking a synthesis of the most recent breakthroughs. This review distinguishes itself by filling these critical knowledge gaps through a comprehensive update of evidence emerging in 2024 and 2025. Specifically, our unique contributions are threefold: First, we systematically integrate the latest high-quality clinical data, including recent large-scale RCTs and studies on specific subtypes like post-stroke depression, to provide a current assessment of therapeutic robustness. Second, we go beyond traditional monoaminergic hypotheses to construct a holistic “neuro-immune-endocrine-gut-brain” mechanistic framework. Notably, we highlight the novel roles of the visual network and sensorimotor network—areas frequently overlooked in previous literature—alongside the vagus-gut axis, offering a fresh perspective on how taVNS remodels whole-brain dynamics. Third, we bridge the gap between basic research and clinical application by critically evaluating emerging machine learning-based predictive models and biomarkers, outlining a roadmap for the precision application of taVNS. Therefore, this review aims to not only synthesize existing clinical evidence to comprehensively evaluate the effectiveness and safety of taVNS in treating depression but also to elucidate its multi-level mechanisms of action and provide theoretical support for accelerating the translation of this technology into personalized clinical practice.

## 2 Methods

### 2.1 Review design

This study is designed as a narrative review aimed at comprehensively synthesizing the current clinical evidence, neurobiological mechanisms, and future research directions of taVNS for MDD. The reporting of this review follows the Scale for the Assessment of Narrative Review Articles (SANRA) guidelines to ensure methodological transparency and quality.

### 2.2 Search strategy

We conducted a systematic search for eligible literature related to taVNS. The search covered English-language databases including PubMed, Web of Science, Embase, and the Cochrane Library, spanning from each database’s inception to October 10, 2025. Search targets included single-arm trials, randomized controlled trials (RCTs), case report and non-randomized controlled trials. The search strategy employed a combination of medical subject headings (MeSH) and free-text keywords related to the intervention and the condition. The specific search terms included: (“transcutaneous auricular vagus nerve stimulation” OR “taVNS” OR “tVNS” OR “auricular vagus nerve stimulation”) AND (“major depressive disorder” OR “MDD” OR “depression” OR “depressive symptoms”).

### 2.3 Study selection

Literature screening began with an initial review based on titles and abstracts. If information was insufficient, full-text articles were obtained for further evaluation. All screening was conducted jointly by the review panel (JZ, DQG and XX). In cases of disagreement, resolutions were reached through discussion or third-party arbitration. Retrieved bibliographic records were imported into EndNote (Analytics, Philadelphia, USA) for automated deduplication and preliminary title/abstract screening. Literature passing the initial screening was imported into Zotero 5.0 (Digital Scholar, Vienna, USA) for full-text review. This study imposed no restrictions on publication date or language.

Inclusion criteria were as follows: 1) Original research articles; 2) Study subjects diagnosed with depression using ICD-10, DSM-IV/V, or CCMD-3; 3) Intervention involving taVNS via electrical stimulation; 4) Study design as randomized controlled trials comparing taVNS with sham stimulation, or single-arm taVNS trials; 5) Studies providing pre- and post-intervention depression scores or treatment response rates, with intervention duration ≥2 weeks. In cases of duplicate publications based on the same dataset, the article with the most comprehensive data reporting was selected.

**Figure 1:**
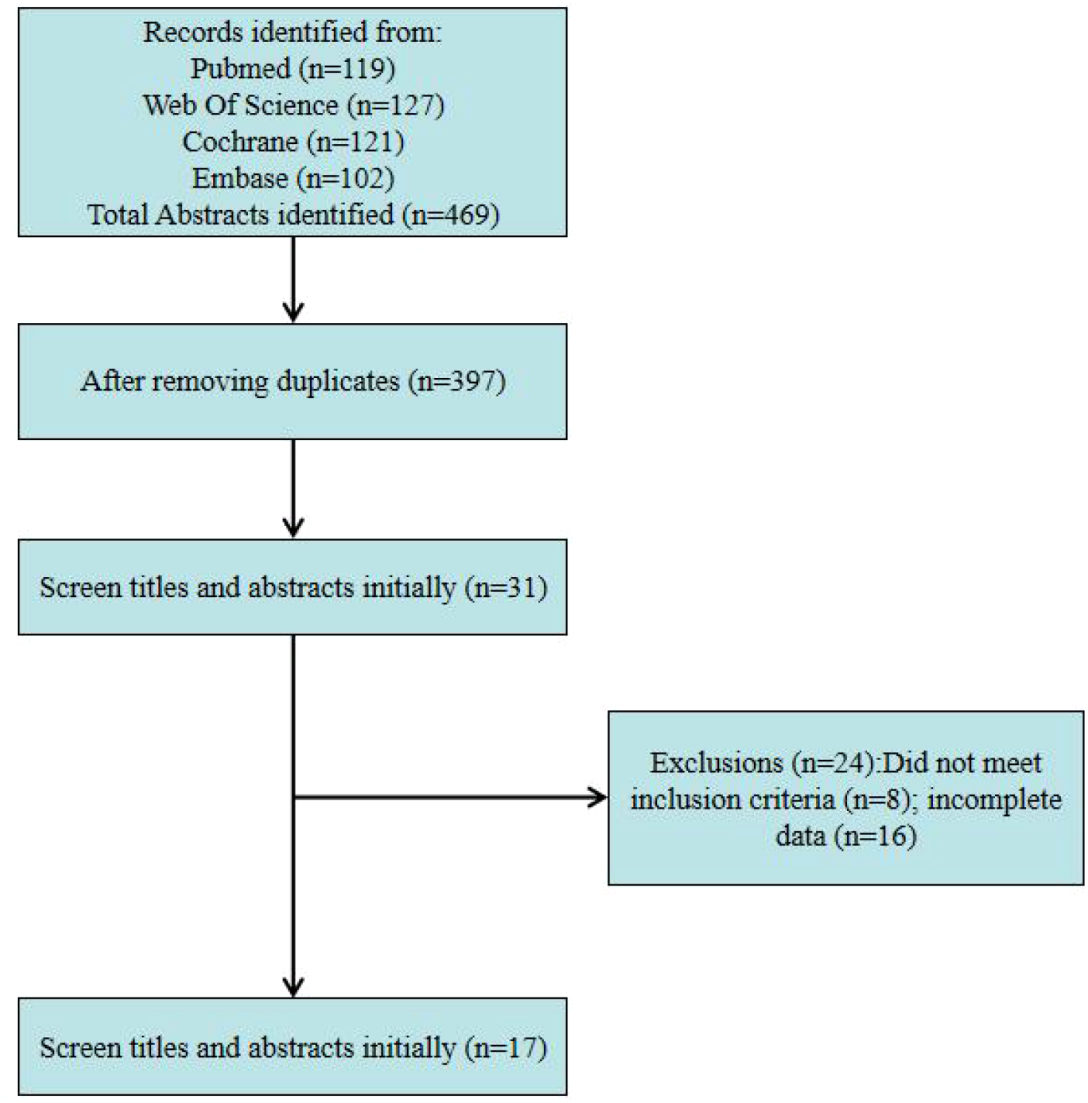
Flow chart of study selection.

Exclusion criteria: 1) Non-primary study types, including systematic reviews, meta-analyses, animal studies, commentaries, letters to the editor, and conference abstracts; 2) Non-electrical trans-temporal nerve stimulation interventions (e.g., acupuncture or auricular acupressure); 3) Failure to report relevant outcome measures; 4) Inaccessible or incalculable study data that remained unavailable despite contacting authors (**Figure 1**).

### 2.4 Data extraction

Data extraction and quality assessment were performed by two independent reviewers (JFS, DQG and JZ). When studies did not report relevant outcome information, we proactively contacted authors to obtain undisclosed details. Extracted information included: 1) First author name, publication year, sample size (control and intervention groups); 2) Baseline characteristics: age, depression type; 3) Intervention method: intervention type, frequency, and duration; 4) Outcome measures: response rate, depression and anxiety severity scores.

Our initial search identified a total of 469 documents. After removing duplicates, 72 studies underwent detailed review. Among these, 36 reports (including full texts and abstracts) were selected for further assessment, with 17 randomized controlled trials ultimately included in the final review. Three reviewers (JZ and XX) extracted data and compiled it into tables.

## 3 A brief introduction to taVNS

### 3.1 The development of taVNS

The development of taVNS represents an innovative integration of traditional wisdom and modern science. Its origins can be traced back to ancient Chinese medical classics such as The Yellow Emperor’s Inner Canon, as well as the “inverted fetus” somatotopic map of the auricle proposed by French physician Paul Nogier in the 1950s (31, 32). These traditional auricular therapies laid the early anatomical and theoretical foundations for taVNS. A critical turning point occurred in the late 20th century with the advent of invasive iVNS (33, 34). Although iVNS was approved for the treatment of drug-resistant epilepsy and depression, its high costs and risks associated with surgical implantation limited its widespread application, thereby generating an urgent demand for non-invasive alternatives (30). In the early 2000s, German scientist Hans Cloe and colleagues achieved a major breakthrough by proposing the revolutionary hypothesis that stimulating the auricular concha, which is innervated by the auricular branch of the vagus nerve, could indirectly activate vagal pathways, based on the correspondence between auricular maps and vagus nerve anatomy (35). This led to the formal naming of taVNS and established it as an emerging field of research. Since then, taVNS research has entered a phase of rapid expansion beginning in the 2010s (6, 36). Advanced imaging techniques such as functional magnetic resonance imaging (fMRI) have not only confirmed that taVNS activates key brain regions including the nucleus tractus solitarius and limbic system, but also elucidated its mechanisms of immune modulation via the “cholinergic anti-inflammatory pathway (37, 38).” The scope of clinical applications has broadened significantly from initial uses in epilepsy and depression to numerous other areas, including anxiety, pain management, cardiovascular diseases, inflammatory conditions, and neurorehabilitation (7, 39, 40).

### 3.2 Auricular vagus nerve

The vagus nerve is the longest and most widely distributed cranial nerve in the human body. It consists of approximately 80% sensory fibers and 20% motor fibers, serving as the primary component of the parasympathetic division of the autonomic nervous system (41). It extensively innervates multiple organs from the neck and chest to the abdomen, playing a key role in regulating critical physiological functions such as heart rate, respiration, digestion, and inflammatory reflexes (42, 43). The auricular branch, a unique portion of the vagus nerve, is its only part distributed on the skin surface. It originates from the superior ganglion and often exhibits anatomical variations or intersections with branches of the facial or glossopharyngeal nerves.

**Figure 2:**
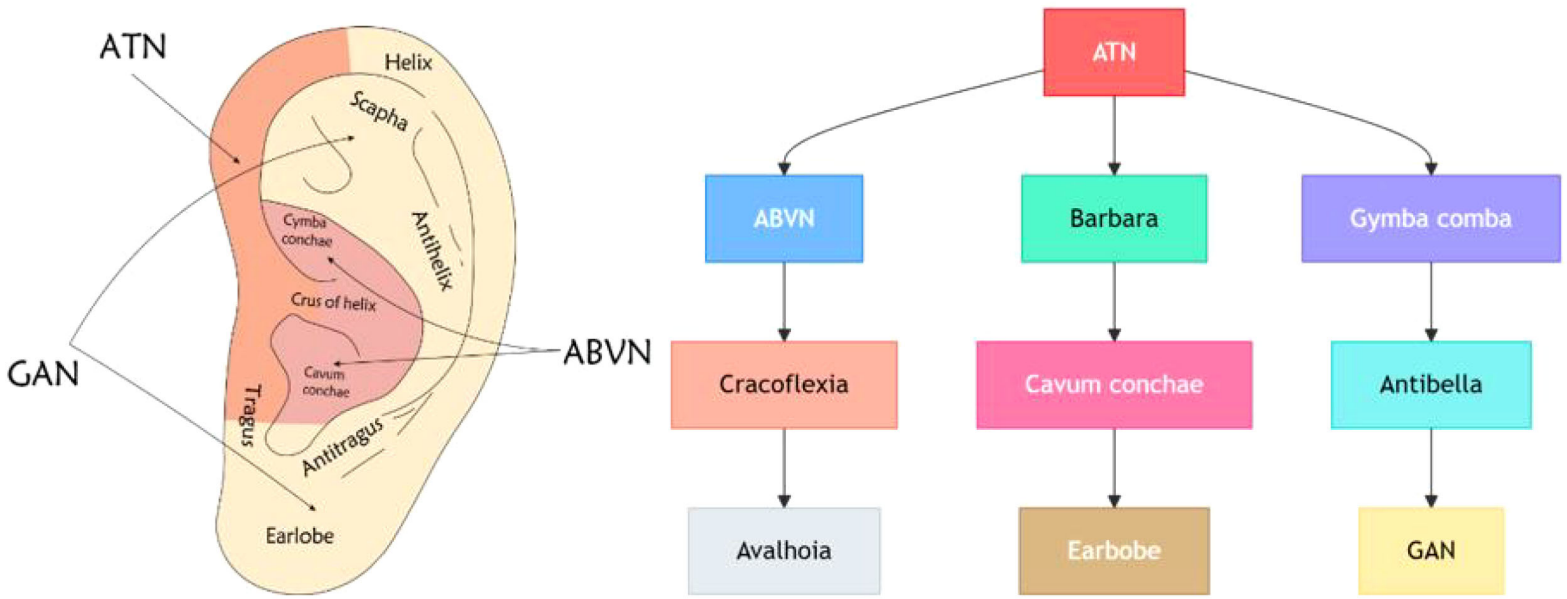
The left side shows different regions of the ear. The right side is a flowchart containing multiple rectangles and arrows. Each rectangle represents a node, and the arrows indicate the relationships between the nodes. ATN, auriculotemporal nerve; GAN, great auricular nerve; ABVN, auricular branch of the vagal nerve.

Its sensory fibers are extensively distributed in the auricular concha and the tragus (31, 44) (**Figure 2**). Anatomically, the cymba conchae is often regarded as the region with the highest density of purely vagal afferent fibers (31, 33). In contrast, the tragus exhibits a more complex innervation pattern, receiving mixed sensory supply from the auricular branch of the vagus nerve as well as the great auricular nerve and the auriculotemporal nerve (33; 45). Despite this mixed innervation, the tragus remains a critical target for stimulation. Seminal work by Badran et al. demonstrated that stimulating the tragus effectively activates the vagal afferent pathway, eliciting robust neuronal activity in the nucleus tractus solitarius (NTS) and downstream limbic regions comparable to concha stimulation (36). Thus, both sites provide viable access points to the vagal network, though via slightly different anatomical substrates.

**Figure 3:**
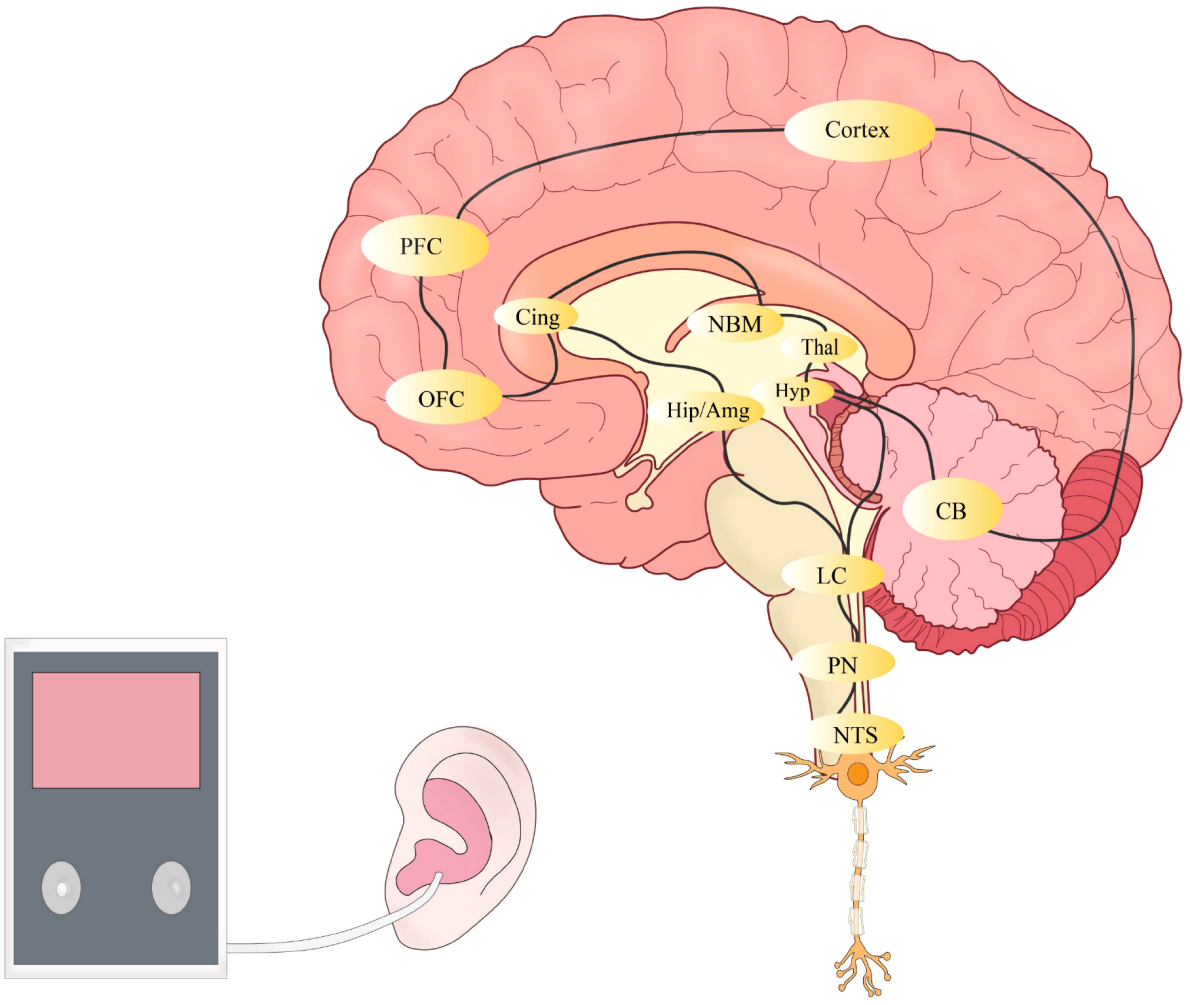
NTS, nucleus tractus solitarius; PN, parabrachial nucleus; LC, locus coeruleus; CB, cerebellum; Thal, thalamus; Hip, hippocampus; Hyp, hypothalamus; Amg, amygdala; NBM, nucleus basalis of meynert; OFC, orbitofrontal cortex; Cing, cingulate cortex; PFC, prefrontal cortex.

Since 80% of the vagus nerve fibers are afferent, they first project to the NTS, which then directly or indirectly connects to multiple brain regions, including the spinal trigeminal nucleus, parabrachial area, dorsal raphe nucleus, locus coeruleus, periaqueductal gray, thalamus, amygdala, and cerebral cortex (39, 44) (**Figure 3**). This extensive neural projection network enables taVNS to achieve multi-level regulation of neural activity and physiological functions.

## 4 The application of TaVNS in depression

### 4.1 Historical development of taVNS for MDD

The development of taVNS for the treatment of depression represents an interdisciplinary innovation process that has evolved from theoretical conception, technological exploration, to in-depth clinical and mechanistic research. This progression fully reflects the deep integration of traditional medical wisdom and modern neuroscience. In 2013, a groundbreaking randomized controlled trial by Hein et al. provided the first evidence supporting the antidepressant effect of taVNS, demonstrating that it significantly reduced Beck Depression Inventory (BDI) scores (46). This study marked the formal entry of taVNS into the stage of evidence-based medical research. Over the following decade, research rapidly deepened and expanded. Multiple studies indicated that taVNS not only effectively improved Hamilton Depression Rating Scale (HAMD) scores (13, 14, 34, 47, 48), but also modulated the DMN and enhanced FC between the amygdala and prefrontal cortex, preliminarily revealing its neural mechanisms (13, 47, 48). As research advanced, the clinical application of taVNS gradually expanded from general MDD populations to various subgroups, including TRD (49, 50), post-stroke depression (PSD) (8) and recurrent depression (51). For instance, studies by Evensen et al. and Li et al. confirmed the clinical efficacy of taVNS in TRD patients and its potential neurochemical mechanisms, such as modulating the GABA/glutamate ratio in the rostral anterior cingulate cortex (rACC) (49, 50). Furthermore, Liu et al. demonstrated that taVNS combined with escitalopram was more effective than monotherapy in treating PSD and significantly increased BDNF levels (8). In recent years, the research focus has shifted toward deeper mechanistic exploration and refined treatment outcome prediction. Emerging studies suggest that taVNS may exert antidepressant effects through multiple mechanisms, including modulating FC in the cortico-striato-pallido-thalamic circuit (15), altering FC between the ventral striatum and precuneus (52), and regulating the nucleus accumbens (NAc)-prefrontal pathway (53).

### 4.2 Critical appraisal and synthesis of clinical evidence

While emerging studies consistently demonstrate the antidepressant efficacy of taVNS, a critical synthesis reveals significant heterogeneity across trials. This variability stems primarily from differences in stimulation dosage, patient subpopulations, and control designs. Understanding these factors is crucial for optimizing clinical translation (**Table 1**).

**Table 1:** Clinical studies of taVNS in MDD.

References	Population	group	Stimulation site	taVNS parameter	Main findings
(46)	37 MDD patients	taVNS (n=18) vs. sham (n=19)	Bilateral outer ear	Frequency 1.5Hz Intensity<600μA Stimulation duration 2 weeks	BDI reduction: 12.6 ± 6.0 (taVNS) vs 4.4 ± 9.9 (sham); 78% patient blinding efficacy
(13)	49 MDD patients (mild/moderate)	taVNS (n=27) vs sham taVNS (n=22)	Left auricular concha	Frequency 20Hz Intensity 4-6mA Stimulation duration 4 weeks	Significant HAMD reduction;DMN FC modulation with limbic regions
(47)	38 MDD patients (mild/moderate)	taVNS (n=17) vs sham taVNS (n=21)	Left auricular concha vs outer ear margin	Frequency 20Hz Intensity 4-6mA Pulse width 0.2 ms Stimulation duration 4 weeks	Significant HAMD reduction; left anterior insula activation predicted treatment response
(48)	49 MDD patients	taVNS (n=18) vs. Sham taVNS (n=16)	Bilateral auricular concha	Frequency 20Hz Intensity 4-6mA Pulse width< 1ms Stimulation duration 4 weeks	Significant HAMD and HAMA reduction; increased amygdala-DLPFC FC
(14)	160 MDD patients	taVNS (n=91) vs. Sham taVNS (n=69)	Auricular concha	Frequency 20Hz Intensity 4-6mA Pulse width 0.2ms Stimulation duration 12 weeks	27% response rate at week 4 vs 0% sham; 80% response rate by week 12
(49)	1 TRD patients	Single-case (n=1)	Bilateral ear concha	Frequency 20Hz Intensity 4-6mA Pulse width<1ms Stimulation duration 8 weeks	Significant HAMD reduction;Increased rACC-DMN FC and decreased GABA/Glu ratios
(54)	41 MDD patients	taVNS (n=20) vs. Sham taVNS (n=21)	Bilateral auricular concha vs. superior scapha (sham)	Frequency 20Hz Intensity 4-6mA Pulse width < 1ms Stimulation duration 4 weeks	Significant HAMD reduction; MH-rACC FC modulation predicts treatment response
(55)	41 MDD patients	taVNS (n=20) vs. Sham taVNS (n=21)	Bilateral auricular concha vs. superior scapha (sham)	Frequency 20Hz Intensity 4-6mA Pulse width< 1ms Stimulation duration 4 weeks	Significant HAMD reduction;NAc-MPFC/rACC FC modulation predicts response
(50)	20 patients with TRD	Single-arm (taVNS)	Left auricular tract	Frequency 25Hz Intensity mean 1.1mA Stimulation duration 4 weeks	significant depression reduction;improved cognitive speed
(51)	25 patients with recurrent depression	Single-arm (taVNS)	Bilateral ear concha	Frequency 20Hz Intensity 4-6mA Pulse width 0.3 ms Stimulation duration 8 weeks	Significant reduction in depression/anxiety scores; FC changes in basal ganglia and sensorimotor networks
(56)	107 MDD patients	taVNS (n=55) vs. Citalopram (n=52)	Bilateral auricular concha	Frequency 20Hz Intensity 4-6mA Pulse width <1ms Stimulation duration 8 weeks	Significant HAMD reduction; similar neurotransmitter modulation
(53)	16 first-episode drug-naive MDD patients vs 16 HCs	MDD group (pre-post design) vs. HC group	Bilateral auricular concha	Frequency 20Hz Intensity 4-6mA Intensity 0.2ms Tolerable intensity (no pain) Stimulation duration 8 weeks	Significant HAMD reduction; increased FC: NAc-dmPFC/vlPFC, vCa-vPFC; decreased FC: vCa-SOG, dCa-cuneus
(57)	22 first-episode drug-naive MDD patients	Single-arm(n=22)	Bilateral auricular concha	Frequency 20Hz Intensity 4-6mA Pulse width < 1ms Stimulation duration 4 weeks	Significant HAMD reduction; decreased ReHo in MCC, PrCG, PoCG, CAL, SMA, PAL, LG
(52)	22 first-episode MDD patients	Single-arm taVNS (n=22)	Bilateral cymba conchae	Frequency 20Hz Intensity 4-6mA Pulse width<1ms Stimulation duration 8 weeks	Significant HAMD reduction; decreased VS-precuneus FC
(8)	80 PSD patients	taVNS+escitalopram (n=40) vs. escitalopram only (n=40)	Left auricular cavum conchae	Frequency 20Hz Intensity 4-6mA Pulse width 1.82 ± 0.4ms Stimulation duration 28 days	Significant HAMD reduction; increased BDNF/CREB/5-HT levels; improved Barthel Index scores
(58)	19 MDD patients (mild/moderate)	Single-arm taVNS (n=19)	Left auricular concha	Frequency 20Hz Intensity 4-6mA Pulse width 0.2 ms Stimulation duration 4 weeks	Significant HAMD reduction and decreased path length post-treatment
(10)	86 MDD patients	taVNS (n=86)	Bilateral auricular concha	Frequency 20Hz Intensity 4-6mA Pulse width < 1ms Stimulation duration 8 weeks	Significant HAMD reduction; 11 predictive FC biomarkers identified; CSPT circuit FCs key predictors

taVNS, Transcutaneous auricular vagus nerve stimulation; FC, Functional connection; TRD, Treatment-resistant depression; HAMD, Hamilton Depression Scale; DMN, Default mode network; VS, Ventral striatum; rACC, Rostral anterior cingulate cortex; DLPFC, Dorsolateral prefrontal cortex; CSPT, Cortico-striatal-pallidal-thalamic; MH, Medial hypothalamus; NAc, Nucleus accumbens; MPFC, Medial prefrontal cortex; MCC, Middle cingulate cortex; PrCG, Left precentral gyrus; PoCG, Postcentral gyrus; CAL, Calcarine cortex; SMA, Supplementary motor area; PAL, Paracentral lobule; LG, Lingual gyrus; dmPFC, Dorsomedial prefrontal cortex; vlPFC, Ventrolateral prefrontal cortex; vCa, Ventral caudate; vPFC, Ventral prefrontal cortex; SOG, Superior occipital gyrus; dCa, Dorsal caudate.

#### 4.2.1 Sources of heterogeneity and methodological quality

The duration of intervention appears to be the most significant factor influencing effect size. Early trials with short durations (≤4 weeks) often reported modest clinical responses. For instance, a landmark study by Rong et al. demonstrated that while the response rate in the taVNS group was only 27% at week 4, it rose substantially to 80% by week 12 (14). This suggests that taVNS induces a cumulative neuroplastic effect rather than an immediate pharmacological-like response. Studies limited to 2-4 weeks may underestimate the true therapeutic potential, potentially explaining conflicting magnitudes of efficacy across the literature (48, 49, 53, 56–58).

The efficacy of taVNS varies across MDD subtypes. In first-episode, drug-naïve patients, taVNS has shown robust modulation of the corticostriatal reward circuits (52, 53). However, in TRD, the response trajectory may differ. While pilot studies indicate feasibility in TRD, these patients often require longer stimulation periods or adjunctive pharmacotherapy to overcome established network rigidity (49, 50). Notably, recent advancements have expanded to specific comorbidities, such as PSD. Liu et al. conducted a high-quality randomized controlled trial showing that taVNS combined with escitalopram significantly outperformed medication alone, suggesting that taVNS may be particularly effective in depression subtypes driven by distinct inflammatory or organic mechanisms (8).

Methodological quality varies significantly. While most studies employed randomized designs, the choice of “sham” stimulation remains a critical confounder. Most trials stimulated the earlobe or superior scapha as a control (13, 14, 46–48, 54, 55). However, recent anatomical evidence suggests that even “non-vagal” ear regions might transmit minor signals via the great auricular nerve to the cervical spinal network, potentially diluting the effect size difference between active and sham groups (33). Furthermore, the lack of active comparators is a notable weakness. Only one study directly compared taVNS with citalopram, finding non-inferiority (56). The majority of existing evidence still relies on small sample sizes, limiting the statistical power to detect subtle biomarker changes.

#### 4.2.2 Clinical implications: towards an optimal protocol

Despite the heterogeneity observed across studies, convergent findings from **Table 1** point towards a set of “optimal” parameters for clinical application. Regarding stimulation sites, both the concha and the tragus are established targets with distinct anatomical characteristics. A critical knowledge gap remains regarding the optimal electrode placement. While the cymba conchae is anatomically recognized as the region with the highest density of purely vagal afferent fibers (31, 33, 44), the tragus—despite its mixed innervation from the great auricular and auriculotemporal nerves—is widely utilized in commercial devices and has demonstrated comparable efficacy in activating brainstem vagal nuclei (36). However, there is currently no consensus on which site yields superior clinical antidepressant outcomes. The lack of rigorous head-to-head comparative trials between concha and tragus stimulation contributes to protocol heterogeneity and hinders the standardization of clinical guidelines.

In terms of frequency, the vast majority of positive trials utilized 20 Hz (8, 13, 14, 51). This frequency aligns with the physiological firing rate of parasympathetic fibers and has been validated to effectively modulate the DMN and anti-inflammatory pathways (5, 13, 38). Stimulation parameters were largely consistent across studies, with a predominant use of current intensities between 4–6 mA and pulse widths under 1 ms (13–15, 47–49, 52–58). Furthermore, to ensure robust clinical remission, a treatment course of at least 8 weeks is recommended (14), while current evidence supports an intensity threshold that is “strong but not painful”, ensuring afferent nerve activation without triggering nociceptive avoidance (13, 53). In conclusion, while taVNS is a promising tool, its transition from an “experimental” technique to a “first-line” therapy requires the implementation of standardized protocols that prioritize adequate treatment duration and precise anatomical targeting (whether concha or tragus). Future high-quality randomized controlled trials must address the sham stimulation dilemma—avoiding active zones or partial innervation pathways (33) —and focus on stratified efficacy across specific MDD subtypes to optimize clinical outcomes and validate predictive biomarkers (15).

## 5 Possible mechanisms of taVNS for treating depression

**Figure 4:**
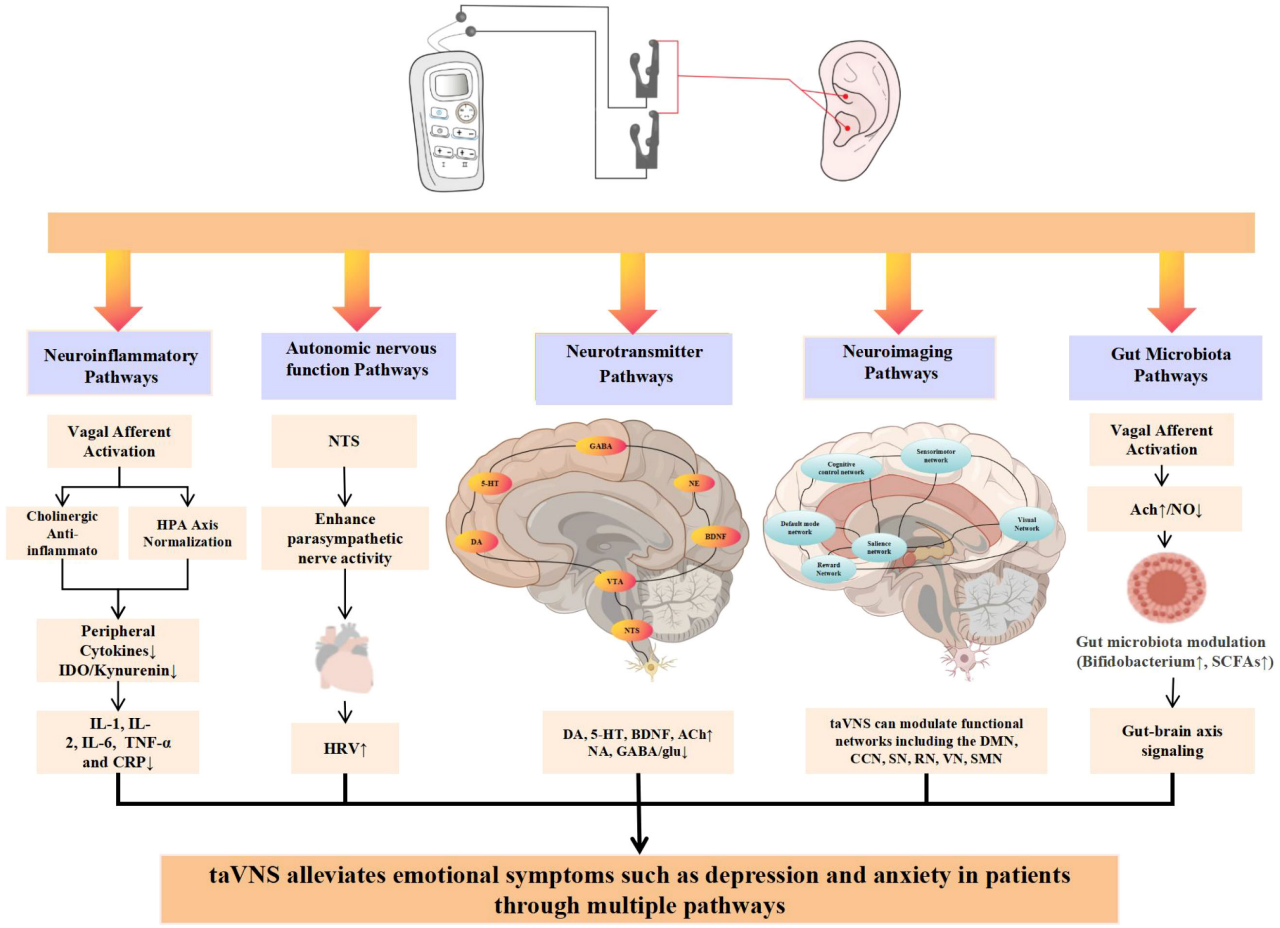
The potential mechanisms of taVNS in treating MDD mainly involve the following five aspects: (1) taVNS can inhibit the release of pro-inflammatory cytokines such as IL-1β, IL-6, and TNF-α, reduce neuroinflammation, and thereby alleviate depressive symptoms; (2) taVNS may enhance parasympathetic nervous activity, regulate autonomic balance, and involve the NTS in central autonomic regulation, promoting emotional stability; (3) taVNS helps restore the functional balance of neurotransmitter systems such as 5-HT, NE, and GABA, exerting a positive regulatory effect on mood and anxiety; (4) taVNS can induce changes in multiple functional networks of the brain, including the default mode network, cognitive control network, salience network, sensorimotor network, and visual network, thereby influencing brain function and structure; (5) taVNS modulates the composition of gut microbiota via the vagus nerve, which in turn regulates central nervous function and emotional state through the gut-brain axis.

The mechanisms of taVNS in treating depression primarily involve five pathways, including neuroinflammatory regulation, autonomic nervous function modulation, neurotransmitter regulation, neuroimaging changes, and the gut microbiota-brain axis (**Figure 4**).

### 5.1 taVNS improves MDD through anti-inflammatory pathways

The etiology of MDD involves multiple biological hypotheses, including dysregulation of monoaminergic neurotransmitter systems, endocrine dysfunction, decreased levels of neurotrophic factors, and overexpression of pro-inflammatory cytokines (17). Among these, abnormal inflammatory factors are considered to play a key role in the pathogenesis of depression (59). A genome-wide association study revealed connections between depression and various genetic and epigenetic networks related to inflammation (60). For example, polymorphisms in cytokines and inflammatory mediators such as IL-1β, TNF-α, and C-reactive protein have been confirmed to correlate with the severity of depression (61). TaVNS exerts significant anti-inflammatory effects by activating the cholinergic anti-inflammatory pathway (61, 62). After stimulating the auricular branch of the vagus nerve, signals are transmitted via afferent pathways to the NTS, which then activates the dorsal motor nucleus of the vagus and the nucleus ambiguous, ultimately regulating the immune function of the spleen through the splenic nerve (63). The core mechanism of this process involves acetylcholine binding to the α7 nicotinic acetylcholine receptor which inhibits the release of pro-inflammatory cytokines from macrophages and simultaneously promotes the expression of anti-inflammatory factors (63). Research indicates that taVNS can significantly reduce inflammation levels in both the peripheral and central nervous systems, ameliorating inflammation-related neural damage (62, 63). This mechanism is particularly important in the treatment of depression, as patients often exhibit a chronic low-grade inflammatory state.

Furthermore, taVNS exerts multi-level regulatory effects on the HPA axis (64). It inhibits the overactivation neurons in the hypothalamic paraventricular nucleus, and improves the rhythmic release of cortisol from the adrenal glands (5). This regulation not only helps alleviate HPA axis hyperactivity but also enhances the function of glucocorticoid receptors, thereby optimizing glucocorticoid signal transduction (65). By synergistically suppressing inflammatory responses and regulating HPA axis function, taVNS provides a unique neuro-immune-endocrine integrative regulatory approach for the treatment of depression. It is especially suitable for patients with depressive subtypes marked by elevated inflammatory biomarkers.

### 5.2 taVNS improves MDD through autonomic nervous system function pathways

Heart rate variability (HRV) refers to the subtle fluctuations in the intervals between consecutive heartbeats. This parameter is finely regulated by neurohumoral systems and serves as a reliable indicator of the dynamic balance between sympathetic and parasympathetic nervous activity (66, 67). As such, HRV has emerged as an essential objective biomarker for evaluating autonomic nervous function and psychological stress levels (67). Numerous studies have confirmed that individuals with depressive disorders typically exhibit lower HRV compared to healthy controls, making HRV an increasingly valuable tool in the auxiliary diagnosis and assessment of depressive states (68). TaVNS is a non-invasive neuromodulation technique that involves the external electrical stimulation of vagal nerve afferent fibers distributed in the auricular region. This stimulation activates vagal afferent pathways, transmitting signals to the NTS in the brainstem. The NTS, a critical hub for visceral sensory integration, further modulates cardiovascular regulatory centers such as the ventrolateral medulla, suppressing excessive sympathetic excitation while enhancing parasympathetic output from the dorsal motor nucleus of the vagus. Consequently, taVNS helps restore autonomic homeostasis (69).

Accumulating evidence demonstrates that taVNS effectively enhances vagal tone and reverses sympathovagal imbalance. This regulatory effect not only contributes to cardiovascular stability but also provides a key physiological basis for taVNS in ameliorating mood disorders and exerting antidepressant effects. Recent studies have shown that taVNS intervention in depressed individuals with sleep deprivation significantly elevates HRV levels, promotes autonomic rebalancing, and markedly alleviates depressive-like behaviors. These findings highlight taVNS as a non-invasive, safe, and promising therapeutic approach for preventing and managing sleep deprivation-related mood disturbances (70). Furthermore, they offer a theoretical foundation and practical direction for developing targeted neuromodulation strategies in high-risk populations.

### 5.3 taVNS improves MDD through neurotransmitter pathways

The pathogenesis of MDD is highly complex, involving dynamic interactions among multiple biological, psychological, and socio-environmental factors. At the biological level, the functional state of neurotransmitter systems is crucial for maintaining chemical balance in the brain. Abnormal levels or disrupted signal transduction in these systems are widely recognized to play a key role in the development and progression of depression (71). Among them, the serotonin system, a core pathway in mood regulation, is closely associated with depressive pathology when dysfunctional (72). TaVNS activates vagal afferent fibers, transmitting signals via the NTS to the dorsal raphe nucleus, thereby modulating the activity of serotonergic neurons. Studies have shown that taVNS treatment significantly reduces plasma 5-HT levels in patients, suggesting that it may restore 5-HT homeostasis through a mechanism similar to that of selective serotonin reuptake inhibitors (56). The mesolimbic dopamine pathway plays a central role in regulating reward and motivational behaviors (73). TaVNS enhances the activity of dopamine neurons in the ventral tegmental area (VTA), promoting dopamine release in the NAc and prefrontal cortex (29). Animal studies further demonstrate that taVNS can reverse chronic social defeat stress-induced social avoidance behavior, an effect that disappears when VTA dopamine neurons are inhibited, indicating that its antidepressant action depends on the functional integrity of the dopamine system (74).

Furthermore, an imbalance between GABAergic inhibition and glutamatergic excitation is considered an important pathological feature of depression (75). TaVNS can enhance GABAergic transmission in the hippocampal region and suppress excessive glutamate release, thereby improving synaptic plasticity and neural circuit function (56). The locus coeruleus (LC)-norepinephrine (NE) system is involved in regulating alertness and stress responses (76). taVNS promotes NE release by activating the vagus nerve–LC pathway, consequently improving cognitive function and emotional stability (56). On the other hand, chronic stress-induced elevated cortisol levels suppress the expression of BDNF (26), while taVNS significantly increases serum BDNF levels, further promoting neural plasticity and neuronal survival, thereby effectively alleviating depressive symptoms (8).

### 5.4 taVNS improves MDD through neuroimaging pathways

Currently, with continuous advances in MRI technology, its application in the research of neuropsychiatric disorders has become increasingly widespread. Neuroimaging studies have shown that the onset and progression of MDD are closely associated with abnormalities in brain functional networks and dysregulation of brain structure coupling. Sheline et al., in the authoritative psychiatric journal Biological Psychiatry, proposed a model hypothesis of a dysregulated “limbic-cortical-striato-pallidal-thalamic circuit” in depression, providing an important theoretical framework for understanding its neuropathological mechanisms. In recent years, neuroimaging research on taVNS for the treatment of MDD has primarily focused on resting-state functional MRI, with key metrics including amplitude of low-frequency fluctuations (ALFF), regional homogeneity (ReHo), and FC. Additionally, one study has begun to incorporate machine learning approaches in this exploration.

#### 5.4.1 taVNS regulates the default mode network

Pathological rumination and excessive self-focus are hallmark cognitive features of MDD, often driven by aberrant hyperactivity within the DMN (77). The DMN is a highly active functional system in the brain during rest, extensively involved in intrinsic cognitive processes such as self-referential thinking, episodic memory retrieval, and emotional regulation (78). This network primarily comprises key brain regions including the medial prefrontal cortex (mPFC), posterior cingulate cortex/precuneus, angular gyrus, and hippocampus (79). Recent studies have indicated that functional abnormalities of the DMN are closely associated with the pathogenesis of MDD (23). Patients with depression often exhibit DMN hyperactivity and aberrant intrinsic FC, particularly within regions such as the mPFC and PCC (80). For instance, Fang et al. conducted a four-week taVNS intervention in patients with mild-to-moderate depression and demonstrated, for the first time, that the therapeutic mechanism of taVNS is related to reduced FC between the DMN and the anterior insula as well as the parahippocampal gyrus (13). This study preliminarily suggests that taVNS may alleviate depressive symptoms by modulating FC between the DMN and limbic system regions.

In a separate case study, Li et al.administered an eight-week combined treatment of taVNS and sertraline to a 55-year-old male patient with a 20-year history of TRD (49). The results indicated significantly enhanced FC between the rACC and the DMN, implying a potential mechanism underlying clinical improvement. Furthermore, Wang et al. revealed that taVNS treatment significantly modulated FC between the left NAc and bilateral mPFC, and this enhanced FC was negatively correlated with reductions in HAMD scores (55). Another study also reported that taVNS can immediately enhance the ALFF in the right precuneus of patients with MDD, while reducing FC between this region and the left middle frontal gyrus, posterior cingulate cortex, and angular gyrus (55). Collectively, these findings suggest that the mechanism by which taVNS ameliorates depressive symptoms may involve not only the modulation of intra-DMN connectivity, but also the regulation of interactions between the DMN and other brain networks. Thus, downregulating DMN hyperactivity and correcting its abnormal coupling with limbic regions may be the neurobiological basis for taVNS in alleviating ruminative symptoms.

#### 5.4.2 taVNS regulates the cognitive control network

Deficits in “top-down” cognitive control lead to the inability to inhibit negative emotions, a core pathogenic mechanism in MDD (81). The cognitive control network (CCN) plays a critical role in emotional processing, top-down emotional regulation, and cognitive memory, among other processes (82). Its core brain regions mainly include the dorsolateral prefrontal cortex (DLPFC), ACC, and parietal cortex (83). A study by Liu et al. showed that after one month of taVNS intervention in patients with MDD, the FC between the right amygdala and the left DLPFC was significantly enhanced, and this enhancement was positively correlated with a reduction in HAMD scores (48). This finding suggests that the mechanism by which taVNS improves clinical symptoms in MDD may be related to its modulation of the CCN. Furthermore, research by Wang et al. indicated that taVNS can also strengthen the FC between the NAc and the anterior cingulate cortex, demonstrating that this stimulation can modulate FC between the reward network (RN) and the CCN, providing important insights into the neural mechanisms of taVNS in treating MDD (55). As a key component of the DLPFC, the middle frontal gyrus also showed significant changes under taVNS modulation, one study in patients with TRD found that 30 minutes of immediate taVNS stimulation significantly enhanced FC between the left orbital area of the middle frontal gyrus and the middle frontal gyrus (84). Another immediate stimulation experiment in MDD patients also observed strengthened FC between the right precuneus and the left middle frontal gyrus (85). These results collectively indicate that the rapid modulatory effects of taVNS on CCN-related brain regions may serve as an important neural basis for its long-term antidepressant effects. By strengthening the connectivity within the CCN and its regulation of limbic structures, taVNS effectively restores the brain’s “top-down” emotional control, thereby improving cognitive function and emotional stability.

#### 5.4.3 taVNS regulates the salience network

The SN is responsible for switching between the DMN and CCN (86). Its dysfunction leads to a “negativity bias” in information processing and difficulty in disengaging from internal distress (87). The SN serves as a central system in the brain for coordinating information, integrating resources, and executing decision-making, playing a critical role in maintaining cognitive flexibility and adaptation to complex environments (88). Its core nodes are primarily located in the ACC and the anterior insula (89). Research has shown that dysfunction of the SN is closely associated with the pathogenesis of MDD (25). A study by Fang et al. demonstrated that taVNS not only significantly alleviates clinical symptoms of MDD but also specifically activates the left insula, an effect which was not observed in the sham stimulation group (47). This suggests that the therapeutic mechanism of taVNS may involve modulation of the SN. Further supporting this, Li et al. reported in a case study on TRD that the clinical improvement induced by taVNS may be related to its regulatory effect on FC between the rostral ACC and the DMN (49). Moreover, research by Tu et al. indicated that after four weeks of taVNS treatment, patients in the real stimulation group showed significantly lower scores on the HAMD scores compared to the sham group (54). Concurrently, FC between the bilateral hypothalamus and the rACC was significantly reduced, and this change in FC strength was correlated with improvements in HAMD scores. These findings not only strengthen the hypothesis regarding the neural mechanisms of taVNS in treating MDD, but also provide important evidence for understanding the role of the SN and related networks in the mechanism of antidepressant treatment. These results suggest that taVNS may correct the “switching failure” of the SN, facilitating the transition from self-referential processing to external cognitive engagement.

#### 5.4.4 taVNS regulates the reward network

Anhedonia and loss of motivation are core symptoms of MDD, stemming primarily from hypoactivation and disconnectivity within the RN (90). The RN is a core neural network in the brain responsible for processing motivation, reward, and pleasure, with key nodes including the NAc, orbitofrontal cortex, and amygdala, among other regions (91). The functional state of this network plays a significant role in the onset and progression of MDD (92). Previous research has shown that the FC between the NAc and the medial prefrontal cortex can effectively predict the degree of clinical symptom improvement in MDD patients following taVNS treatment (55). Furthermore, evidence suggests that taVNS exerts its therapeutic effects by reducing FC between the right NAc and the right DLPFC. This mechanism indicates that taVNS may alleviate depressive symptoms by modulating corticostriatal circuit function (53). Further supporting this, a study by He et al. demonstrated that taVNS significantly enhances FC between the striatum and the precuneus, suggesting that its clinical benefits in MDD may stem from reversing functional abnormalities in these brain regions (52). In addition, research by Sun et al. compared neural activity between patients with TRD and healthy controls, finding that the TRD group exhibited significantly higher ALFF in the left orbital area of the middle frontal gyrus (84). After taVNS treatment, ALFF values decreased notably in this region as well as in the right middle frontal gyrus. Together, these findings indicate that taVNS modulates neural activity within the RN and related brain areas, which may represent a key neurobiological mechanism through which it improves clinical outcomes in MDD. Therefore, taVNS appears to rescue the “reward deficiency” state by enhancing corticostriatal connectivity, offering a targeted mechanism for alleviating anhedonia.

#### 5.4.5 taVNS regulates the sensorimotor network

Psychomotor retardation and somatic symptoms are common in MDD, reflecting functional anomalies in the sensorimotor network (SMN) (93).The SMN is one of the key functional networks in the brain, playing a central role not only in sensory integration and motor execution but also extensively involved in higher-order functions such as executive control, emotion regulation, and cognitive processing (94, 95). This network primarily encompasses multiple brain regions including the precentral gyrus, postcentral gyrus, supplementary motor area, posterior parietal cortex, and cerebellum (93). Previous studies have shown that taVNS treatment in patients with recurrent MDD significantly reduces FC between the globus pallidus and the SMN. Moreover, changes in FC between the right globus pallidus and the left inferior parietal lobule, as well as between the left globus pallidus and the right postcentral gyrus, were negatively correlated with changes in HAMD scores (51). Another study focusing on first-episode drug-naïve MDD patients found that after taVNS intervention, ReHo was significantly decreased in several brain regions, including the left precentral gyrus, left postcentral gyrus, left supplementary motor area, and left paracentral lobule. Additionally, changes in ReHo in the right median cingulate cortex/left supplementary motor area were positively correlated with changes in HAMD scores (56). Furthermore, a machine learning study by Sun et al. indicated that FC between the cerebellum and the globus pallidus may serve as an important neuroimaging marker for predicting the efficacy of taVNS treatment in MDD patients (15). This implies that taVNS may alleviate psychomotor retardation and somatic discomfort by modulating the excitability and connectivity of the sensorimotor system.

#### 5.4.6 taVNS regulates the visual network

Abnormal visual attention bias towards negative stimuli is an early cognitive marker of depression, involving dysregulation of the Visual Network (VN) (96). The VN, primarily composed of brain regions such as the occipital lobe, calcarine sulcus, cuneus, and lingual gyrus, plays a critical role in visual information processing and visuospatial cognition (97). Previous studies have indicated that the neuropathological mechanisms of MDD exhibit stage-specific characteristics throughout its onset and progression (98). In recent years, the role of the VN in MDD has garnered increasing attention (99). Research by Zhang et al. demonstrated that the therapeutic effect of taVNS in first-episode depression is associated with the modulation of FC between the striatum and the occipital cortex, suggesting that regulating FC between the reward system and the visual network may contribute to alleviating depressive symptoms (53). Furthermore, Sun et al. employed an innovative double-nested and leave-one-out cross-validation approach to develop a predictive model for taVNS treatment efficacy, revealing that FC centered in the right superior occipital gyrus serves as a key neuroimaging feature in predicting treatment response to taVNS (15). In summary, taVNS may mitigate negative attentional bias by remodeling the functional architecture of the VN and its coupling with the reward system.

### 5.5 taVNS improves MDD through gut microbiota pathways

The gut microbiota pathway represents a key integrative loop through which taVNS improves MDD (100). Gut microbiota directly or indirectly regulate the dynamic balance of digestive, neural, immune, neuroendocrine, and enteric nervous system functions through multiple pathways (101–103). Recent studies have revealed a close association between gut dysbiosis and the pathogenesis of MDD, with the vagus nerve serving as a central mediator in this process (100, 104).

**Figure 5:**
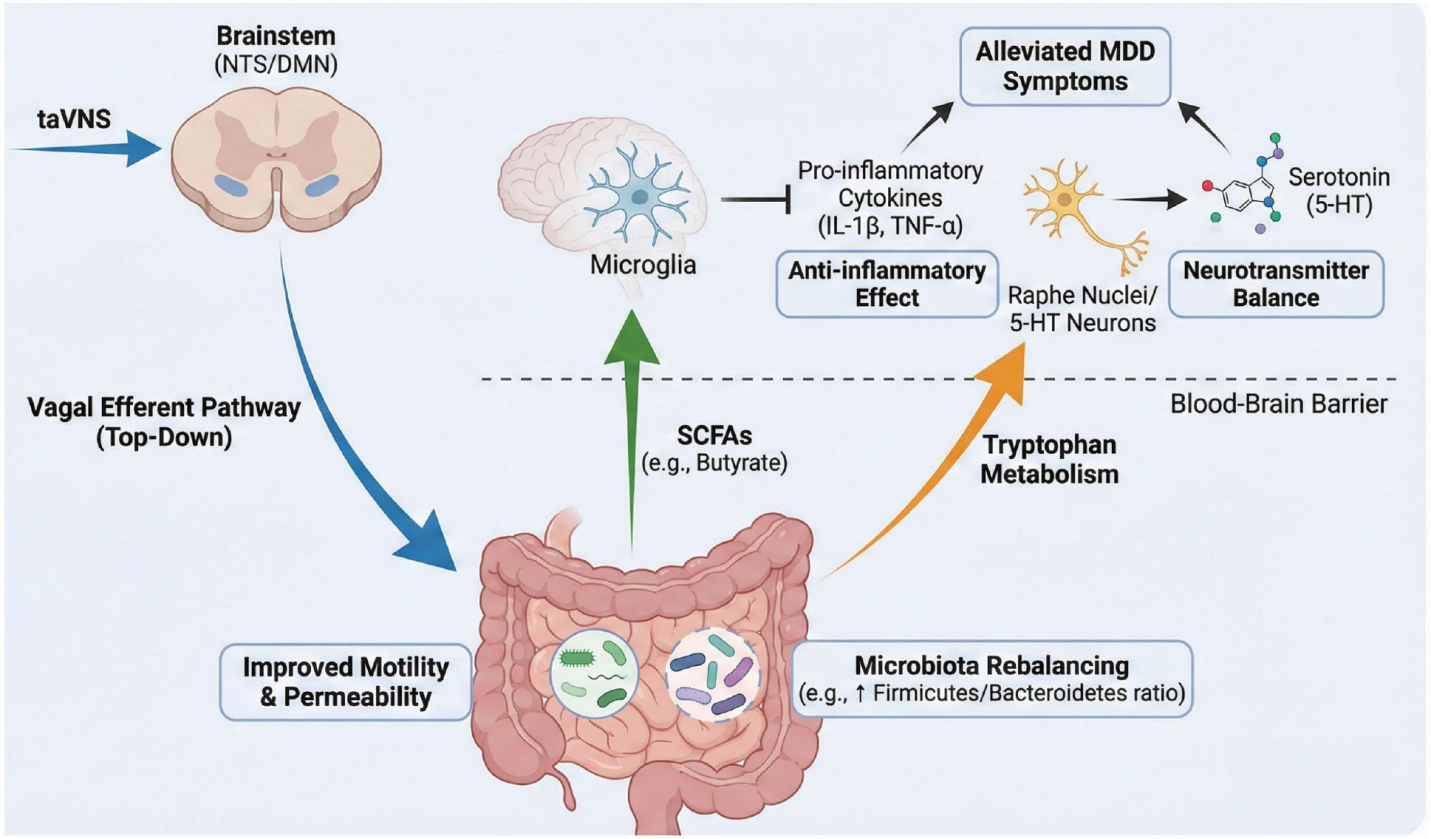
Schematic representation of the integrative mechanism of taVNS via the Gut-Brain Axis. The figure illustrates the bidirectional “closed-loop” modulation of taVNS in treating MDD: (1) Top-Down Modulation (Blue Arrow): taVNS signals are transmitted via the brainstem through the vagal efferent pathway to the gut. This stimulation improves gastrointestinal motility and reduces permeability, leading to Microbiota Rebalancing. (2) Bottom-Up Mediation: The rebalanced microbiota exert therapeutic effects via two key metabolic pathways that cross the Blood-Brain Barrier.

taVNS modulates gut–brain interactions via bidirectional “top-down” and “bottom-up” mechanisms (105). On one hand, taVNS activates the dorsal motor nucleus of the vagus nerve, regulating gastrointestinal motility and reducing intestinal permeability, thereby improving the gut microenvironment and correcting microbiota imbalances commonly observed in depression, such as altered Firmicutes/Bacteroidetes ratios (105). Clinical evidence from patients with constipation-predominant irritable bowel syndrome indicates that taVNS can effectively modify gut microbiota composition, which correlates with alleviation of depressive symptoms (106). On the other hand, taVNS restores the abundance of short-chain fatty acid (SCFA)-producing bacteria (e.g., Fecalibacterium), increasing metabolites such as butyrate and propionate (105). These metabolites can cross the blood–brain barrier and act as histone deacetylase inhibitors, suppressing microglial activation and the release of pro-inflammatory cytokines, thereby directly linking gut metabolism with central anti-inflammatory pathways (105). Additionally, by reducing systemic inflammation and restoring microbial balance, taVNS helps shift tryptophan metabolism toward serotonin synthesis, increasing 5-HT availability and aligning with neurotransmitter regulatory mechanisms (100). In summary, taVNS operates through an integrated “Neuro-Immune-Endocrine-Gut-Brain” circuit: it suppresses systemic inflammation and HPA axis hyperactivity, thereby optimizing the gut environment; in turn, the restored gut microbiota provides essential metabolic substrates that jointly support neuroplasticity and emotional stability (100, 105) (**Figure 5**).

## 6 Limitations and future directions

**Figure 6:**
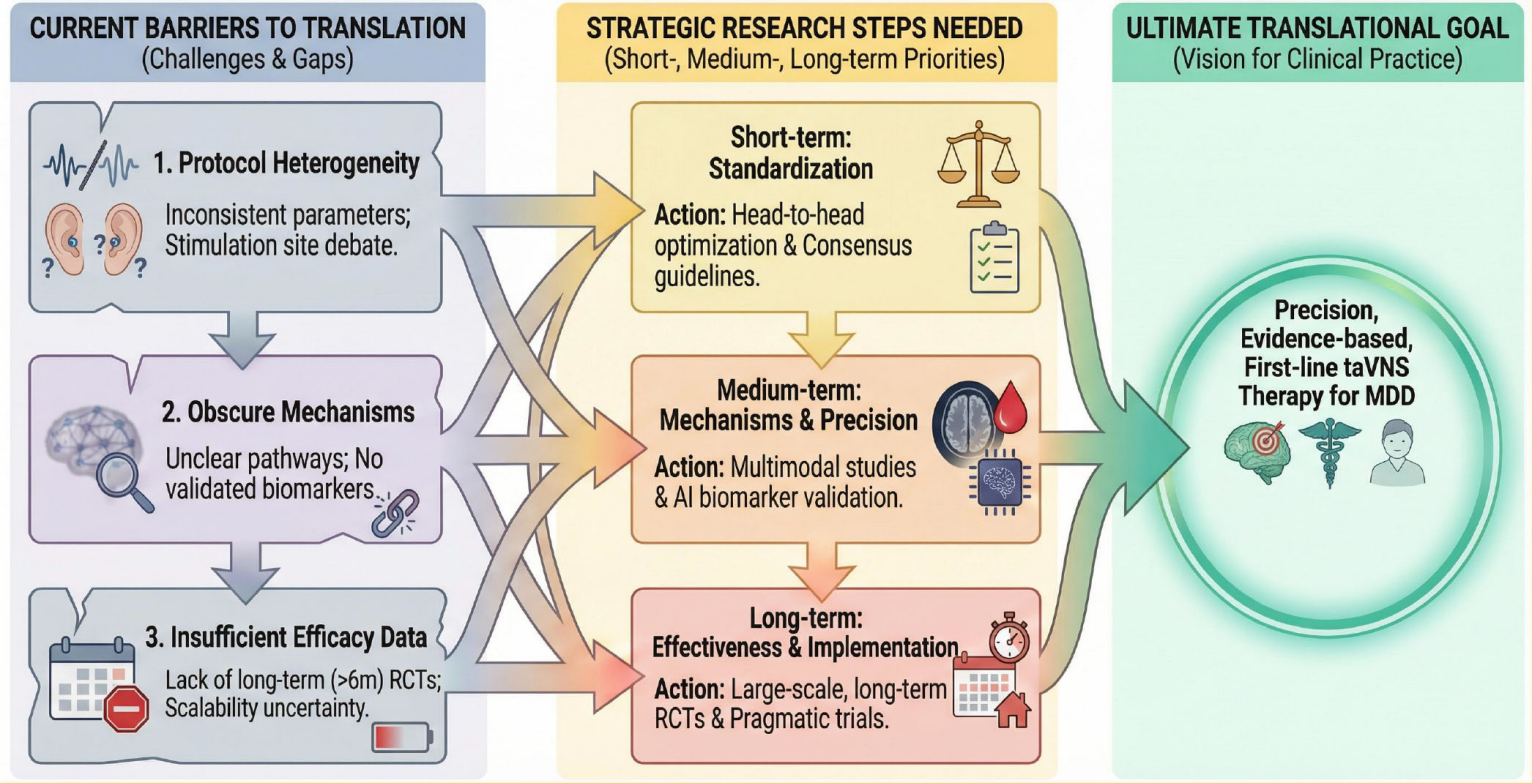
Conceptual framework for the clinical translation of taVNS in MDD.Left: Key barriers to translation. Middle: Strategic research steps organized by short-, medium-, and long-term priorities to address these barriers. Right: The ultimate goal of establishing taVNS as a precise, first-line therapy.

While taVNS demonstrates significant potential as a non-invasive intervention for MDD, the transition from promising experimental findings to robust clinical application is currently hindered by methodological heterogeneity and a lack of large-scale evidence. To bridge this gap, we propose a hierarchical research agenda prioritized into short-, medium-, and long-term phases (**Figure 6**).

### 6.1 Short-term priorities: standardization and validation

The immediate priority is to resolve methodological inconsistencies that currently hinder valid cross-study comparisons. This necessitates a threefold effort: (1) establishing consensus on optimal stimulation parameters, with current evidence supporting 20 Hz frequency and a pulse width of <1 ms for modulating the default mode network and anti-inflammatory pathways, while future studies must rigorously define the dose-response relationship and compare the anatomical efficacy of cymba conchae versus tragus stimulation given their distinct innervation patterns; (2) refining sham controls by developing truly inert protocols or using active controls with different frequencies (e.g., 1 Hz vs. 20 Hz) to isolate specific vagal effects from placebo responses, as stimulating non-vagal sites like the earlobe may still activate the great auricular nerve; and (3) replicating promising neuroimaging biomarkers, such as functional connectivity changes in the corticostriatal circuit and visual network, in independent cohorts to establish reliable biomarkers for objective treatment monitoring.

### 6.2 Medium-term priorities: mechanisms and precision medicine

Following parameter standardization, research should deepen our understanding of how and for whom taVNS works most effectively. First, multimodal mechanistic integration is crucial: future RCTs should adopt an integrated “neuro-immune-endocrine-gut-brain” framework and concurrently measure fMRI network changes, inflammatory cytokines, HPA-axis markers, and gut microbiota composition to holistically elucidate how taVNS drives neuroplasticity through systemic physiological loops. Second, subtype-specific optimization must be addressed: given the high heterogeneity of depression, research should stratify efficacy across distinct subtypes, such as TRD and PSD, and tailor stimulation protocols for patients with specific physiological profiles, such as elevated inflammation or reward system deficits. Third, although recent studies have employed machine learning to identify potential neuroimaging predictors, such as functional connectivity in the corticostriatal circuit (15), a major gap exists in the absence of validated, reproducible predictive biomarkers for clinical use. Current findings are largely derived from small-sample exploratory studies without independent validation cohorts. Consequently, clinicians lack objective tools to identify ‘responders’ prior to treatment initiation. Future research must prioritize the validation of multimodal biomarkers to enable precision psychiatry.

### 6.3 Long-term priorities: effectiveness and implementation

Perhaps the most significant barrier to establishing taVNS as a first-line therapy is the insufficiency of long-term efficacy data. The vast majority of existing RCTs are limited to acute intervention periods of 4 to 8 weeks (14). Data extending beyond 6 months are virtually non-existent, leaving a critical knowledge gap regarding the durability of remission, the necessity of maintenance sessions, and long-term safety. To bridge this gap, future multicenter trials must incorporate follow-up periods exceeding 6 months to assess relapse prevention and sustained benefits. The ultimate goal is to establish taVNS as a scalable, first-line therapeutic option. This requires three coordinated advances: first, conducting large-scale, multicenter effectiveness trials with sufficiently powered sample sizes to detect small-to-moderate effects, which must include long-term follow-up (>6 months) to assess durability of remission and relapse prevention. Second, performing rigorous comparative effectiveness research through non-inferiority or superiority trials that directly evaluate taVNS against current standards, such as pharmacotherapy and established neuromodulation techniques, to definitively position it within the treatment algorithm. Third, capitalizing on the inherent portability of taVNS by developing wearable, user-friendly devices for home-based implementation, integrated with mobile health technologies for real-time symptom and physiological monitoring, thereby revolutionizing long-term disease management and accessibility.

## 7 Conclusions

TaVNS has emerged as a promising non-invasive neuromodulation technique for the treatment of MDD. Current evidence, though largely derived from small-sample and short-term studies, consistently indicates that taVNS can significantly alleviate depressive symptoms, modulate aberrant brain network activity, and restore neurochemical and autonomic balance. Its mechanisms of action are multifaceted, involving anti-inflammatory pathways, autonomic nervous system regulation, neurotransmitter modulation, functional network reorganization, and gut–brain axis communication. However, limitations such as methodological heterogeneity, a lack of large-scale randomized controlled trials, and insufficient long-term follow-up hinder the clinical translation and standardization of taVNS. Future research should prioritize multicenter, large-sample studies, optimize stimulation parameters, explore predictive biomarkers, and investigate taVNS efficacy in specific MDD subtypes and combination therapies. Through integrated multimodal approaches and advanced technologies, taVNS holds great potential to become a safe, effective, and accessible therapeutic option for patients with depression.
