# Correction: Transcutaneous auricular vagus nerve stimulation for major depressive disorder: current evidence and future research directions

**DOI:** 10.3389/fpsyt.2026.1855399

**Published:** 2026-04-27

**Authors:** Jifei Sun, Deqiang Gao, Jing Zhang, Hongwei Liu, Yuan Zhou, Chenjie Ma, Xiaojian Zhang, Shuqing Liu, Jingxue Zhao, Chunbo Hao, Xue Xiao

**Affiliations:** 1 Department of Neurology, Shunyi Hospital, Beijing Hospital of Traditional Chinese Medicine, Beijing, China; 2 Guang’anmen Hospital, China Academy of Chinese Medical Sciences, Beijing, China; 3 Department of Pain Management, Beijing Fengtai You’anmen Hospital, Beijing, China; 4 Department of Psychiatry and Psychology, Beijing Tsinghua Changgung Hospital, Beijing, China

**Keywords:** gut-brain axis, major depressive disorder, narrative review, neuroimaging, neuromodulation, transcutaneous auricular vagus nerve stimulation

In the published article, there was an error in the affiliations. Affiliation 1 for authors “Jifei Sun, Hongwei Liu, Yuan Zhou, Chenjie Ma, Xiaojian Zhang, Shuqing Liu, and Chunbo Hao” was erroneously given as “Department of Neurology, Shunyi Hospital, Beijing University of Chinese Medicine, Beijing, China”. The correct affiliation is “Department of Neurology, Shunyi Hospital, Beijing Hospital of Traditional Chinese Medicine, Beijing, China”.

The original version of this article has been updated.

